# Efficacy of 2 different fibroblast growth factor receptor-inhibitors in a patient with extrahepatic cholangiocarcinoma harboring an FGFR2 mutation: a case report

**DOI:** 10.1093/oncolo/oyae294

**Published:** 2025-05-08

**Authors:** Ilianna Galli-Vareia, Petr Szturz, Ioannis A Voutsadakis, Nicolas Villard, Georgia Tsoumakidou, Mapi Fleury, Gabriela Herrera, Francois Fasquelle, Sebastien Godat, Antonia Digklia

**Affiliations:** Department of Oncology, Centre Hospitalier Universitaire Vaudois, Lausanne 1011, Switzerland; Department of Oncology, Centre Hospitalier Universitaire Vaudois, Lausanne 1011, Switzerland; Algoma District Cancer Program, Sault Area Hospital, Sault Ste Marie, ON P3E 2C6, Canada; Division of Clinical Sciences, Section of Internal Medicine, Northern Ontario School of Medicine, Sudbury, ON P3E 2C6, Canada; Department of Radiology, Centre Hospitalier Universitaire Vaudois, Lausanne 1011, Switzerland; Department of Radiology, Centre Hospitalier Universitaire Vaudois, Lausanne 1011, Switzerland; Department of Oncology, Centre Hospitalier Universitaire Vaudois, Lausanne 1011, Switzerland; Department of Oncology, Centre Hospitalier Universitaire Vaudois, Lausanne 1011, Switzerland; Department of Pathology, Centre Hospitalier Universitaire Vaudois, Lausanne 1011, Switzerland; Department of Gastroenterology, Centre Hospitalier Universitaire Vaudois, Lausanne 1011, Switzerland; Department of Oncology, Centre Hospitalier Universitaire Vaudois, Lausanne 1011, Switzerland

**Keywords:** cholangiocarcinoma, next-generation sequencing, targeted therapy, *FGFR2* mutation

## Abstract

Cholangiocarcinoma (CCA) is a type of cancer with few effective systemic therapies. Elucidation of the molecular landscape of the disease from genomic studies based on next-generation sequencing (NGS) has contributed to the introduction of new targeted therapies. One of these treatments consists of a class of small molecules that target members of the fibroblast growth factor receptors (FGFRs) family of receptor tyrosine kinases. We report here on a patient with a cholangiocarcinoma bearing an FGFR2 mutation. The patient was treated with 2 different FGFR inhibitors, as the first caused ocular toxicity. She obtained clinical benefits from both. This case illustrates the efficacy of FGFR inhibitors on cholangiocarcinoma with specific point mutations.

**Key points**

- To our best knowledge, this is the first report of a very successful treatment with 2 consecutive FGFR2 inhibitors.
- Molecular pathological result can guide precision treatment for cholangiocarcinoma patients.
- FGFR2 mutations may also be sensible to fibroblast growth factor receptor inhibitors.

## Patient story

A 68-year-old Caucasian woman was diagnosed with type IIIb cholangiocarcinoma, according to the BISMUTH–CORLETTE classification, stage IIIC. She underwent an exploratory laparotomy through a Makuuchi incision, a large right hepatectomy in segments I and IV, cholecystectomy, direct porto-portal reconstruction, and bilio-digestive anastomosis. The pathologic staging according to the TNM 8th edition was pT4 pN1 (2/6) L1 V1 Pn1 G2, with positive margins.

The patient received adjuvant treatment with capecitabine for 4 cycles, interrupted due to peritoneal progression when the patient was treated with first-line palliative chemotherapy of cisplatin and gemcitabine.^1^ The treatment was complicated by grade 2 thrombocytopenia that necessitated a 40% dose reduction and multiple treatment interruptions. Due to peritoneum-limited disease, the patient underwent 3 cycles of pressurized intraperitoneal aerosol chemotherapy (PIPAC) with oxaliplatin. Due to peritoneal progression, second-line chemotherapy with the FOLFIRI regimen^2^ was initiated.

Further progression was documented in November of 2019. Two cycles of FOLFOX^3^ were attempted, complicated by grade 3 thrombocytopenia and disease progression. In the absence of a standard third-line treatment option, a genomic NGS analysis was performed on the Ion GeneStudio S5 System platform (Ion Torrent, Thermo Fisher Scientific) using the Ion AmpliSeq Custom Cancer Hotspot Panel IPA (custom version). This genomic panel covers 218 hotspot regions of 52 genes and identified an FGFR2 exon 7 mutation c.755C > G (p.Ser252Trp). Interestingly, no mutation in KRAS, TP53, and CDKN2A driver genes and those involved in the DNA repair pathway were detected.

Due to a lack of appropriate studies and approved drugs at that time, treatment was started with the fibroblast growth factor receptor (FGFR) tyrosine kinase inhibitor erdafitinib at a dose of 5 mg once daily based on the data of Park et al^4^ and Bahleda et al^5^ as part of a compassionate use program (pre-approval access JNJ-41756493), stabilizing the disease. The treatment was interrupted when a grade 3 ocular toxicity (dry eye syndrome) was confirmed by ophthalmological evaluation after complaints of blurred vision limiting self-care, an adverse effect that affected approximately 25% of patients receiving this drug.^6^ The patient was treated by iridectomy and was started on vitamin A and artificial tears. The adverse effect subsided 1 month after treatment interruption.

**Figure 1.**
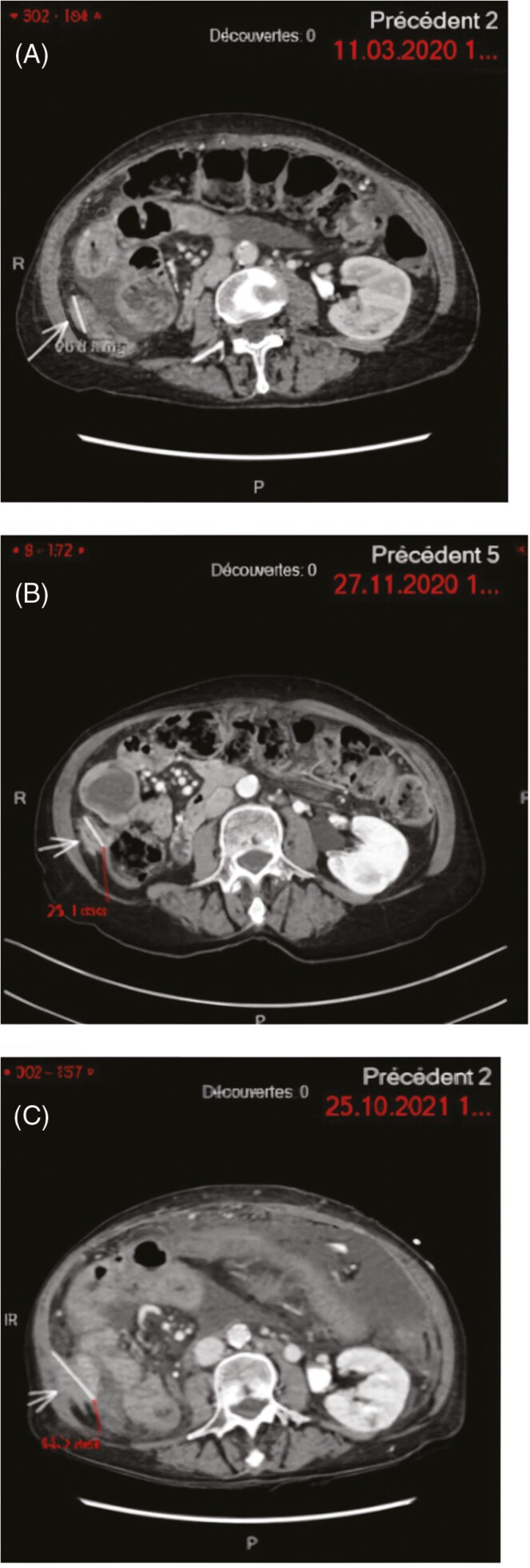
Peritoneal carcinosis target-lesion. MRI images showing the trajectory of the disease during specific time points. (A) March 11, 2020: Erdafitinib starting point, (B) November 27, 2020: Erdafitinib discontinuation due to toxicity—stable disease, (C) October 24, 2021: Pemigatinib starting point—progressive disease.

The patient received no treatment till a radiological and clinical progression. At that time another FGFR inhibitor, pemigatinib was initiated, based on the preliminary data of Abou-Alfa et al^7^ The initial dose was 4.5 mg once daily for 2 out of 3 week cycles, instead of the standard 13.5 mg dose due to the patients reduced performance status (PS) with an Eastern Cooperative Oncology Group (ECOG) grade of 2. A clinical response was achieved after 4 weeks of pemigatinib therapy associated with regression of ascites and ECOG improvement to 0. The drug was discontinued after 9 months of treatment after a discussion with the patient due to the development of hyperbilirubinemia secondary to biliary obstruction from biliary sludge and free-floating tumor debris, requiring multiple interventions. Although only stable disease was observed as the best response based on Response evaluation criteria in solid tumors (RECIST) criteria on follow-up CT scans, the response was maintained (Figure 1).

## Molecular tumor board

Our patient harbored a somatic mutation at the exon 7 (c.755C > G (p.Ser252Trp)) of FGFR2. This specific mutation of the extracellular domain of the protein has also been described in Apert Syndrome (or acrocephalosyndactyly type I), a congenital autosomal dominant syndrome, one of the most common craniosynostosis syndromes, with a prevalence of 1 in 65 000 individuals. Besides craniosynostosis and syndactyly, Apert syndrome patients commonly present midface retrusion, palatal and dental abnormalities, strabismus, hearing loss, and feeding difficulties. The codon 252 mutation observed in our patient is associated with a higher prevalence of cleft palate in patients with Apert syndrome.^8^ Another frequent genetic point mutation in the *FGFR2* gene in individuals with the syndrome is on the neighboring codon 253 (exon 7 of *FGFR2* gene 758 C > G, p.Pro253Arg (P253R)) and has also been described in patients with CCA.^9^ The Apert syndrome is part of developmental receptor tyrosine kinasepathies, congenital malformation syndromes resulting from mutations in receptor tyrosine kinases of several families, including, the *FGFR*, *EGFR*, *TRK*, *PDGFR*, and *VEGFR* family.^10^ In cancer, *FGFR2* p.Ser252Trp (S252W) mutations were reported in endometrial carcinomas.^11^ When transduced in NIH 3T3 fibroblasts, *FGFR2* p.Ser252Trp produced anchorage-independent growth. Endometrial cell lines with the specific mutation were inhibited by FGFR inhibitor PD173074 in vitro, through attenuation of activity of downstream signaling protein FRS2, constitutively phosphorylated by the mutant *FGFR2*.^12^ In cholangiocarcinoma, an International Cancer Genome Consortium (ICGC) study showed FGFR2 mutations in 12 of 417 (2.9%) analyzed patients.^13^ One of the 12 patients had the *FGFR2* p.Ser252Trp mutation. This patient had fluke-associated intrahepatic cholangiocarcinoma and was alive at the time of the report, 38.9 months after diagnosis.^12^ In another genomic series with exclusively iCCA patients, *FGFR2* mutations were observed in 18 of 412 (4.4%) patients.^14^ None of these patients had the *FGFR2* p.Ser252Trp mutation. In the iCCA cohort from the American Association of Cancer Research (AACR) project GENIE (public version 13.1), 49 of 964 analyzed patients (5.1%) had FGFR2 mutations, none being at codon 252.^15^

Our patient was treated off-label according to the paradigm of CCAs with *FGFR2* fusions or rearrangements despite having a mutation in the gene. Since the time of her treatment, there have been ongoing studies, that evaluated several selective FGFR tyrosine kinase inhibitors in the context of multiple *FGFR* alterations, including mutations (Table 1). Our patient obtained a long-lasting benefit from 2 different FGFR targeting therapies not approved for her specific *FGFR2* alteration. Pemigatinib, infigratinib, and futibatinib have been approved by the US Food and Drug Administration (FDA) for the treatment of patients with previously treated advanced cholangiocarcinoma with *FGFR2* fusion or rearrangement. Pemigatinib was also EMA-approved. Pemigatinib was evaluated in a phase I/II open-label study in 128 patients with advanced solid tumors (FIGHT-101).^16^ The study included an escalation and an expansion cohort. Overall, about half of the patients presented hyperphosphatemia and fatigue. Other adverse events observed included dry mouth, alopecia, and stomatitis. The most frequently observed severe (grade ≥ 3) adverse events were pneumonia (10%), fatigue (8%), and hyponatremia (8%). Hyperphosphatemia was managed with diet and phosphate binders, but dose modifications were also necessary. The recommended phase II dose selected was 13.5 mg once daily. In the dose expansion cohort (group 2), 4 patients with cholangiocarcinoma were treated with pemigatinib, with 1 achieving a partial response (PR) at a dose of 9 mg daily and the response was maintained.^16^ Overall, across cancers, patients with *FGFR* rearrangements or fusions had a response rate (RR) of 25% and patients with *FGFR* mutations had a RR of 23.1%.^16^

**Table 1.** Treatment-related adverse events occurring in more than 10% of all anti-FGFR treated patients.

	Erdafitinib^6^	Pemigatinib^7^	Futibatinib^21^	Infigratinib^19^	All FGFR inhibitors
	*N* = 187	*N* = 146	*N* = 103	*N* = 108	*N* = 544
Any adverse event	173 (93%)	Not reported (NR)	102 (99%)	104 (96%)	379 (70%)
Hyperphosphatemia	119 (64%)	81 (55%)	88 (85%)	80 (74%)	368 (68%)
Stomatitis	69 (37%)	47 (32%)	21 (20%)	55 (51%)	192 (35%)
Dry mouth	78 (42%)	42 (29%)	31 (30%)	23 (21%)	174 (32%)
Alopecia	33 (18%)	67 (46%)	34 (33%)	35 (32%)	169 (31%)
Dysgeusia	48 (26%)	55 (38%)	19 (18%)	28 (26%)	150 (28%)
Diarrhea	38 (20%)	53 (36%)	29 (28%)	19 (18%)	139 (26%)
Fatigue	24 (13%)	47 (32%)	26 (25%)	31 (29%)	128 (24%)
Dry skin	49 (26%)	23 (16%)	28 (27%)	22 (20%)	122 (22%)
Dry eye	25 (13%)	31 (21%)	17 (17%)	34 (31%)	107 (20%)
Decreased appetite	42 (22%)	35 (24%)	13 (13%)	16 (15%)	106 (19%)
Hand–foot syndrome	20 (11%)	22 (15%)	22 (21%)	35 (32%)	99 (18%)
Nausea	30 (16%)	36 (25%)	12 (12%)	11 (10%)	89 (16%)
Constipation	26 (14%)	20 (15%)	17 (17%)	10 (9%)	73 (13%)
Asthenia	52 (28%)	19 (13%)^b^	NR	NR	71 (13%)
Vomiting	19 (10%)	14 (10%)	20 (19%)^b^	14 (13%)	67 (12%)
Nail toxicity	20 (11%)^a^	11 (8%)^c^	16 (16%)	19 (18%)^c^	66 (12%)
Arthralgia	NR	22 (15%)	10 (10%)	31 (29%)	63 (12%)

^a^Onycholysis.

^b^All cause adverse events.

^c^Nail discoloration.

The efficacy of pemigatinib on CCAs harboring FGFR alterations was further evaluated in the phase II study FIGHT-202.^7^ Patients with CCA and disease progression after at least 1 previous treatment was assigned to 1 of 3 cohorts: patients with *FGFR2* fusions or rearrangements (*n* = 107), patients with other FGF/FGFR alterations (*n* = 20), or patients with no FGFR alterations (*n* = 18). The primary endpoint was the objective response rate (ORR) among those with fusions or rearrangements. All patients received at least 1 dose of pemigatinib. After a median follow-up of 17.8 months, an overall response rate (ORR) of 35.5% was observed in the 107 patients with fusions or rearrangements. Median progression-free survival (PFS) was 6.9 months and median overall survival (OS) was 21.1 months. No patients with other FGF/FGFR alterations achieved responses and 40% had stable disease. Their median PFS and OS were 2.1 months and 6.7 months, respectively. Among the 20 patients, no patient had the FGFR2 S252W mutation. The most prevalent *FGFR2* mutation was *FGFR2* C382R, observed in 4 patients.^17^ Three of the 4 patients achieved stable disease with pemigatinib. The same C382R mutation was reported in a patient with metastatic CCA treated with pemigatinib.^18^ This patient achieved a complete response to treatment by MRI evaluation, associated with a complete functional response as confirmed by PET-scanning ongoing at 10 months. The most common all-grade adverse event was hyperphosphatemia in 60% of patients. Most frequent grade ≥ 3 adverse events were hypophosphatemia, arthralgia, stomatitis, hyponatremia, abdominal pain, and fatigue, without treatment-related deaths.^7^

Infigratinib has been approved in the same setting, having obtained an ORR of 18.8% and a disease control rate of 83.3%.^19^ In a phase II trial, 5 patients with *FGFR2* mutations were included and all had stable disease.^20^ Moreover, futibatinib was granted an accelerated approval for treatment of locally advanced/metastatic iCCA with an *FGFR2* gene rearrangement or fusion, based on the phase II FOENIX-CCA2 trial.^21^ In this single-arm trial, 103 iCCA patients harboring FGFR2 fusion/rearrangements and disease progression after 1 or more prior treatments received futibatinib at 20 mg once daily. At a median follow up of 17 months, the confirmed ORR was 42% and the median PFS was 9.7 months, with a 1-year PFS rate of 40%. The median OS was 21.7 months, with a 1-year OS rate of 72%.

Another FGFR inhibitor, erdafitinib has not been granted approval for biliary tract carcinoma, but it has an indication for FGFR-altered urothelial carcinoma and has been studied in biliary tract cancers. The first phase I erdafitinib trial enrolled 187 patients with advanced or refractory solid tumors, including 11 CCA patients.^5^ All 11 patients had FGFR alterations (including 1 FGFR2 and 2 FGFR3 mutations). The ORR for CCK patients was 3 of 11 (27%, all PR). The response duration was 11.4 months. Among the 3 responding patients, 2 had fusions and one had a FGFR2 mutation. The 2 other patients with FGFR3 mutations and had progressive disease as their best response (Table 1).^5^ The most common adverse events were hyperphosphatemia, dry mouth and stomatitis, most of them of grade 1 or 2. Skin changes, nail and eye disorders (dry eye up to 29% in maximal dosing) were also common. Grade 3 events or higher were infrequent, the main one being anemia in 17 patients. Adverse events were considered the main cause of death in 9 patients, including 2 bleeding complications. Table 1 summarizes the different adverse profiles among these FGFR inhibitors.

More recently, a phase II trial of tinengotinib, a large spectrum multi-kinase inhibitor targeting FGFRs 1-3, Aurora A/B, Janus kinase (JAK)1 and 2 and vascular endothelial growth factor receptors has been reported. Interestingly, tinengotinib has shown promising clinical benefit in 48 patients with FGFR2 fusion CCA after prior FGFR inhibition, as well as in patients with non-fusion FGFR alterations or wild type, with manageable toxicity.^22^ Lirafugratinib (RLY-4008), another FGFR2i showed to be efficacious in a phase 1 trial without causing clinically significate hyperphosphatemia or diarrhea.^23^

Multikinase inhibitors with activity against FGFR2 kinase have shown efficacy in isolated cases of patients with cholangiocarcinoma with FGFR2 mutations.^24^ Two patients with CCA progressing on chemotherapy exhibited PR to pazopanib and lenvatinib lasting 11 and 11.6 months, respectively. Both patients had FGFR2 mutations, an F276C point mutation the one, and a 2 amino acids inframe deletion and single amino acid insertion in the juxta membrane domain of the protein on the other.

Given the diverse effect of point mutations in the conformation of FGFR proteins, it is unlikely that all mutations in FGFR2 have the same sensitivity to the targeted agents. Further collaborative research will be needed to identify specific FGFR mutations, most sensitive to FGFR inhibitors. Moreover, many cases with fusions or rearrangements initially sensitive to FGFR inhibition acquire treatment resistance due to distinct mutations under treatment pressure.^25^ Whether this will be a mechanism of resistance in FGFR-mutated patients remain to be studied. Several points are worth noting in our patient. This is the first reported case of FGFR inhibitors in CCA with the FGR2 S252W mutation, suggesting clinical efficacy. In addition, it is only one of few studies reporting pemigatinib efficacy in any FGFR2 mutant CCA. Another notable side is that, despite erdafitinib discontinuation for ocular adverse effects, there was no radiologic progression for 11 months. Absence of other alterations (TP53, KRAS, and CDKN2A) may explain the long survival.^14^

Finally, similar FGFR inhibitors, do not necessarily lead to similar side effects. While FGFR alterations are common in iCCAs, they were also found in extrahepatic CCAs as in our case report, demonstrating the importance of molecular analysis in therapeutic decision-making.

## Patient update

**Figure 2.**
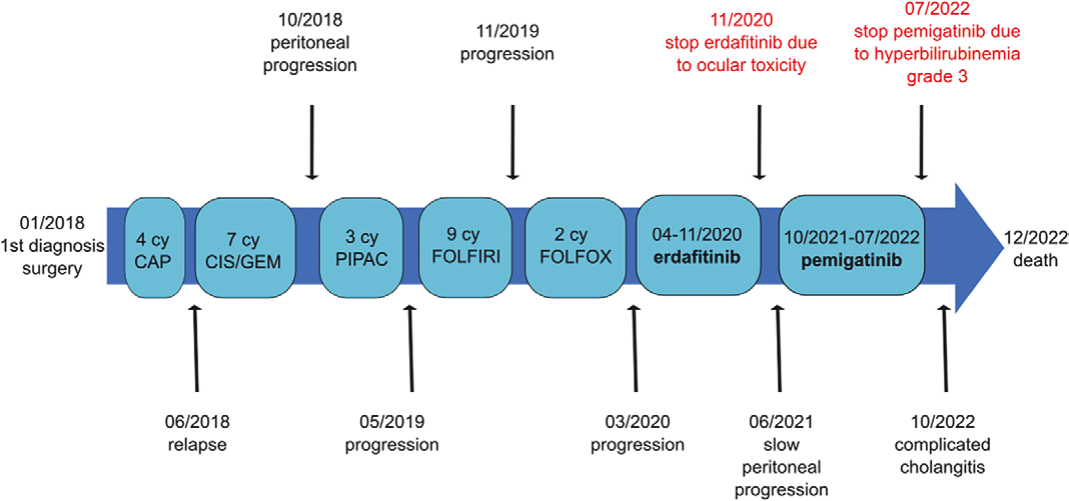
Timeline showing disease course summary. In January 2018, the diagnosis was made and the patient was operated. Afterward, after 4 cycles of adjuvant capecitabin, the patient has relapsed and a first line of cisplatine—gemcitabine has been started. In October 2019, after a peritoneal progression, 3 cycles of PIPAC have been realized. From May 2019 to March 2020, a second and third line of chemotherapy were administrated. From April to November 2020, erdafitinib has been given, with disease stabilization, having to be terminated because of ocular toxicity. After a period of slow peritoneal progression, pemigatinib was introduced in October 2021. It has been terminated in July 2022 because of grade 3 hyperbilirubinemia. The disease was once more stabilized. After a complicated cholangitis in October 2022, the patient finally passed away in December 2022.

The patient ultimately developed severe cholangitis and sepsis, due to intraluminal stent obstruction by sludge. She succumbed to this infectious complication. No signs of disease progression were evident at the time of her death. Figure 2 summarizes the disease course.

## Data Availability

Data available on request from the authors.

## References

[1] Valle J , Wasan H, Palmer DH, et al Cisplatin plus gemcitabine versus gemcitabine for biliary tract cancer. N Engl J Med. 2010;362:1273-1281. https://doi.org/10.1056/nejmoa0908721 20375404

[2] Caparica R , Lengelé A, Bekolo W, Hendlisz A. FOLFIRI as second-line treatment of metastatic biliary tract cancer patients. Autops Case Rep. 2019;9:e2019087. https://doi.org/10.4322/acr.2019.087. PMID: 31528622; PMCID: PMC6738847 31528622 PMC6738847

[3] Lamarca A , Palmer DH, Wasan HS, et al; Advanced Biliary Cancer Working Group. Advanced Biliary Cancer Working Group. Second-line FOLFOX chemotherapy versus active symptom control for advanced biliary tract cancer (ABC-06): a phase 3, open-label, randomised, controlled trial. Lancet Oncol. 2021;22:690-701. https://doi.org/10.1016/S1470-2045(21)00027-9 33798493 PMC8082275

[4] Park JO , Feng Y-H, Chen Y-Y, et al Updated results of a phase IIa study to evaluate the clinical efficacy and safety of erdafitinib in Asian advanced cholangiocarcinoma (CCA) patients with FGFR alterations. J Clin Oncol. 2019;37:4117-4117. https://doi.org/10.1200/jco.2019.37.15_suppl.4117

[5] Bahleda R , Italiano A, Hierro C, et al Multicenter phase I study of erdafitinib (JNJ-42756493), oral pan-fibroblast growth factor receptor inhibitor, in patients with advanced or refractory solid tumors. Clin Cancer Res. 2019;25:4888-4897. https://doi.org/10.1158/1078-0432.ccr-18-3334 31088831

[6] Loriot Y , Necchi A, Park SH, et al; BLC2001 Study Group. Erdafitinib in locally advanced or metastatic urothelial carcinoma. N Engl J Med. 2019;381:338-348. https://doi.org/10.1056/NEJMoa1817323 31340094

[7] Abou-Alfa GK , Sahai V, Hollebecque A, et al Pemigatinib for previously treated, locally advanced or metastatic cholangiocarcinoma: a multicentre, open-label, phase 2 study. Lancet Oncol. 2020;21:671-684. https://doi.org/10.1016/s1470-2045(20)30109-1 32203698 PMC8461541

[8] Benmiloud S , Chaouki S, Atmani S, Hida M. Apert syndrome. Pan Afr Med J 2013;14:66. https://doi.org/10.11604/pamj.2013.14.66.2178 23565313 PMC3617707

[9] Li Y , Ma D, Sun Y, et al Apert Syndrome With FGFR2 758 C > G mutation: a Chinese case report. Front Genet. 2018;9:181. https://doi.org/10.3389/fgene.2018.00181 29868125 PMC5966571

[10] McDonell LM , Kernohan KD, Boycott KM, Sawyer SL. Receptor tyrosine kinase mutations in developmental syndromes and cancer: two sides of the same coin. Hum Mol Genet. 2015;24:R60-R66. https://doi.org/10.1093/hmg/ddv254 26152202 PMC4572000

[11] Dutt A , Salvesen HB, Chen TH, et al Drug-sensitive FGFR2 mutations in endometrial carcinoma. Proc Natl Acad Sci USA. 2008;105:8713-8717. https://doi.org/10.1073/pnas.0803379105 18552176 PMC2438391

[12] Pollock PM , Gartside MG, Dejeza LC, et al Frequent activating FGFR2 mutations in endometrial carcinomas parallel germline mutations associated with craniosynostosis and skeletal dysplasia syndromes. Oncogene. 2007;26:7158-7162. https://doi.org/10.1038/sj.onc.1210529. Epub 2007 May 21. PMID: 17525745; PMCID: PMC2871595 17525745 PMC2871595

[13] Jusakul A , Cutcutache I, Yong CH, et al Whole-genome and epigenomic landscapes of etiologically distinct subtypes of cholangiocarcinoma. Cancer Discov. 2017;7:1116-1135. https://doi.org/10.1158/2159-8290.CD-17-0368 28667006 PMC5628134

[14] Boerner T , Drill E, Pak LM, et al Genetic determinants of outcome in intrahepatic cholangiocarcinoma. Hepatology. 2021;74:1429-1444. https://doi.org/10.1002/hep.31829 33765338 PMC8713028

[15] Pugh TJ , Bell JL, Bruce JP, et al; AACR Project GENIE Consortium, Genomics and Analysis Working Group. AACR Project GENIE: 100,000 Cases and Beyond. Cancer Discov. 2022;12:2044-2057. https://doi.org/10.1158/2159-8290.CD-21-1547 35819403 PMC9437568

[16] Subbiah V , Iannotti NO, Gutierrez M, et al FIGHT-101, a first-in-human study of potent and selective FGFR 1-3 inhibitor pemigatinib in pan-cancer patients with FGF/FGFR alterations and advanced malignancies. Ann Oncol. 2022;33:522-533. https://doi.org/10.1016/j.annonc.2022.02.001 35176457 PMC11961695

[17] Silverman IM , Hollebecque A, Friboulet L, et al Clinicogenomic analysis of FGFR2-rearranged cholangiocarcinoma identifies correlates of response and mechanisms of resistance to pemigatinib. Cancer Discov. 2021;11:326-339. https://doi.org/10.1158/2159-8290.CD-20-0766 33218975

[18] Hempel L , Lapa C, Dierks A, et al A new promising oncogenic target (p.C382R) for treatment with pemigatinib in patients with cholangiocarcinoma. Ther Adv Med Oncol. 2022;14:17588359221125096. https://doi.org/10.1177/17588359221125096 36188486 PMC9520138

[19] Javle M , Roychowdhury S, Kelley RK, et al Infigratinib (BGJ398) in previously treated patients with advanced or metastatic cholangiocarcinoma with FGFR2 fusions or rearrangements: mature results from a multicentre, open-label, single-arm, phase 2 study. Lancet Gastroenterol Hepatol. 2021;6:803-815. https://doi.org/10.1016/S2468-1253(21)00196-5 34358484

[20] Javle M , Lowery M, Shroff RT, et al Phase II Study of BGJ398 in patients with FGFR-altered advanced cholangiocarcinoma. J Clin Oncol. 2018;36:276-282. https://doi.org/10.1200/JCO.2017.75.5009 29182496 PMC6075847

[21] Goyal L , Meric-Bernstam F, Hollebecque A, et al; FOENIX-CCA2 Study Investigators. Futibatinib for FGFR2-rearranged intrahepatic cholangiocarcinoma. N Engl J Med. 2023;388:228-239. https://doi.org/10.1056/NEJMoa2206834 36652354

[22] Milind MJ , et al Efficacy and safety results of FGFR1-3 inhibitor, tinengotinib, as monotherapy in patients with advanced, metastatic cholangiocarcinoma: results from phase II clinical trial. J Clin Oncol. 2024;42:434-434. https://doi.org/10.1200/JCO.2024.42.3_suppl.434

[23] Schönherr H , Ayaz P, Taylor AM, et al Discovery of lirafugratinib (RLY-4008), a highly selective irreversible small-molecule inhibitor of FGFR2. Proc Natl Acad Sci USA. 2024; 121: e2317756121. https://doi.org/10.1073/pnas.2317756121 38300868 PMC10861881

[24] Bitzer M , Spahn S, Babaei S, et al Targeting extracellular and juxtamembrane FGFR2 mutations in chemotherapy-refractory cholangiocarcinoma. NPJ Precis Oncol. 2021;5:80. https://doi.org/10.1038/s41698-021-00220-0 34480077 PMC8417271

[25] Krook MA , Lenyo A, Wilberding M, et al Efficacy of FGFR inhibitors and combination therapies for acquired resistance in FGFR2-fusion cholangiocarcinoma. Mol Cancer Ther. 2020;19:847-857. https://doi.org/10.1158/1535-7163.MCT-19-0631 31911531 PMC7359896

